# Work-related medical rehabilitation in patients with mental disorders: the protocol of a randomized controlled trial (WMR-P, DRKS00023175)

**DOI:** 10.1186/s12888-021-03181-7

**Published:** 2021-05-03

**Authors:** Miriam Markus, Nina Gabriel, Markus Bassler, Matthias Bethge

**Affiliations:** 1 Institute of Social Medicine and Epidemiology, University of Lübeck, Ratzeburger Allee 160, 23562 Lübeck, Germany; 2 Rehabilitation Sciences and Health Services Research, Institute for Social Medicine, Nordhausen University of Applied Sciences, Weinberghof 4, Nordhausen, 99734 Germany

**Keywords:** Work-related medical rehabilitation, Effectiveness, Work ability, Return to work, Mental disorders

## Abstract

**Background:**

Various rehabilitation services and return-to-work programs have been developed in order to reduce sickness absence and increase sustainable return-to-work. To ensure that people with a high risk of not returning to work can participate in working life, the model of work-related medical rehabilitation was developed in Germany. The efficacy of these programs in patients with mental disorders has been tested in only a few trials with very specific intervention approaches. To date, there is no clear evidence of the effectiveness of work-related medical rehabilitation implemented in real-care practice.

**Methods/design:**

Our randomized controlled trial will be conducted in six rehabilitation centers across Germany. Within 15 months, 1800 patients with mental disorders (300 per rehabilitation center) will be recruited and assigned one-to-one either to a work-related medical rehabilitation program or to a conventional psychosomatic rehabilitation program. Participants will be aged 18–60 years. The control group will receive a conventional psychosomatic rehabilitation program without additional work-related components. The intervention group will receive a work-related medical rehabilitation program that contains at least 11 h of work-related treatment modules. Follow-up data will be assessed at the end of the rehabilitation and 3 and 12 months after completing the rehabilitation program. The primary outcome is a stable return to work. Secondary outcomes cover several dimensions of health, functioning and coping strategies. Focus groups and individual interviews supplement our study with qualitative data.

**Discussion:**

This study will determine the relative effectiveness of a complex and newly implemented work-related rehabilitation strategy for patients with mental disorders.

**Trial registration:**

German Clinical Trials Register (DRKS00023175, September 29 2020).

**Supplementary Information:**

The online version contains supplementary material available at 10.1186/s12888-021-03181-7.

## Background

The 12-month prevalence of mental disorders in the German general population is about 30%. Contrary to public perception, this prevalence rate has been remarkably stable since the 1970s [1, 2]. However, the consequences of illness are becoming more visible: sickness absence [3, 4], health-related early retirement [5] and recognized severe disabilities [6] due to mental disorders have increased significantly in recent years. A study in general practitioners’ practices has shown that people with mental disorders have twice as many days of sickness absence as people with chronic somatic illnesses [7].

In many countries, rehabilitation services are available to support patients with chronic diseases in order to enable work participation [8]. In Germany, roughly one-sixth of inpatient rehabilitation programs for working-age people are provided to those with mental disorders (total: 16.7%; men: 12.7%; women: 20.7%) [9]. Depression, adjustment disorders, somatoform disorders and anxiety disorders are the most frequent disorders treated [10].

Due to the adverse consequences of mental illness for work participation, researchers have increasingly used work participation outcomes to appraise the effects of rehabilitation programs in recent years. In a randomized controlled trial, Hillert and colleagues have investigated the effects of a work-related medical rehabilitation (WMR) approach in a sample of patients with long-term sickness absence and unemployed patients. Participants in the intervention group were exposed to real work environments by completing part-time internships outside the rehabilitation center over a period of 4 weeks. This work exposure was accompanied by behavioral therapy in the rehabilitation department. One year after the rehabilitation program, participants in the intervention group returned to work significantly more often and reported more positive job-related attitudes [11]. A study by Beutel and colleagues examined the effects on work participation outcomes of a similar program but followed a psychodynamic instead of a behavioral approach. These authors reported shorter sickness absences and more positive attitudes towards work for the intervention group [12]. Nieuwenhuijsen et al. [13] conducted a Cochrane Review to investigate whether occupational interventions in depressed patients, in addition to clinical interventions, promote return to work. They analyzed data from three randomized controlled trials with 251 patients. In these trials, concrete action plans for work problems were developed, but the scope and focus of the interventions varied. In two of the three studies, the employer or the company medical officer was contacted. The duration of sickness absence during the follow-up period was reduced by almost half a standard deviation compared with the control group (standardized mean difference: − 0.40; 95% confidence interval: − 0.66 to − 0.14). The quality of the evidence was downgraded from high to moderate due to the low number of patients included in the meta-analysis. In summary, the national and international studies show that additional work components that are closely linked to conventional psychosomatic treatment can achieve more favorable work participation outcomes.

Rehabilitative services provided by the German Pension Insurance aim at work participation and the avoidance of disability pensions. The increasingly stronger orientation towards these goals is reflected in the dissemination of WMR programs. For the implementation of such programs, pension insurances have developed joint recommendations concerning target group, content and scope. These recommendations are summarized in the WMR guidelines [14–17]. WMR programs regularly comprise a diagnostic assessment that compares job demands and patients’ work capacity and offers therapeutic interventions such as work hardening and work-related functional capacity training, work-related psychosocial groups and intensified social counseling (see also the Methods section) [15, 18–20]. The aim of WMR is to improve the work participation of persons at high risk of not returning to work by taking greater account of individual work demands and the required skills. Possible indicators of a high risk of not returning to work are, for example, long-term or repeated sick leave and an unfavorable subjective poor return-to-work expectation. Representative administrative data indicated that 57% of the patients with mental disorders undergoing rehabilitation in 2013 may have an increased risk of not returning to work due to long-term sickness absence before rehabilitation, unemployment at the time of applying for rehabilitation or reduced work capacity in the last job as determined by a socio-medical evaluation [14, 16]. Up to now, the evidence regarding the efficacy of WMR in mental disorders has been based on only two randomized controlled trials, and these trials have tested a very specific approach (work exposure and accompanying psychotherapeutic groups). There is no clear evidence of the effectiveness of the WMR implemented in real-care practice based on the WMR guidelines.

### Objectives

We hypothesize that WMR improves stable return to work 12 months after rehabilitation (primary outcome) compared with conventional psychosomatic rehabilitation (CPR). Moreover, we expect more favorable secondary outcomes in patients treated with WMR.

### Trial design

The study is a randomized controlled trial with two parallel groups. Participants are randomly assigned to a WMR or CPR group in a one-to-one ratio. Focus groups and individual interviews supplement our study with qualitative data.

## Methods

### Study setting

Participants will receive either a WMR or CPR program in one of six rehabilitation centers located in Germany (Rehazentrum Oberharz in Clausthal-Zellerfeld, Reha-Zentrum Seehof in Teltow, Fachklinik Aukrug, Klinik am Hainberg in Bad Hersfeld, Celenus Parkklinik in Bad Bergzabern and MediClin Bliestal Kliniken in Blieskastel). The rehabilitation programs will be provided as inpatient programs. Participation was approved either by the Federal German Pension Insurance or a regional pension insurance agency. In both programs, interventions will be performed by rehabilitation physicians, psychologists, physiotherapists, sports therapists, social workers, occupational therapists and other health professionals.

The duration of the rehabilitation program is initially determined by the pension insurance agency (usually about 5 weeks). The rehabilitation center and the patient can agree on an extension of the program. By request, the patient may stop the rehabilitation program ahead of schedule.

### Eligibility criteria

Patients are aged 18–60 years and have chronic mental disorders. Only patients with a need for WMR (i.e. with severe restrictions in work ability and a higher risk of not returning to work) will be included in the study. Patients have requested rehabilitation due to mental disorders interfering with work ability. Need for rehabilitation was acknowledged by a registered doctor and approved by the pension insurance agency. After approval, the pension agencies assign patients to the participating rehabilitation centers. The need for WMR is identified in the participating rehabilitation center by the SIMBO-C, a standardized screening instrument [21–24]. Patients are eligible if they have a risk score of at least 27 out of 100 points.

### Treatment

#### Control

Participants of the control group will receive a CPR program according to current treatment standards and guidelines for the rehabilitation of mental disorders. CPR programs last approximately 5 weeks. The duration of daily therapy amounts to 3 or 4 h. Following a multimodal approach, CPR programs include psychotherapy, psychoeducation, relaxation training, sports and exercise therapy, health education, expressive arts therapy, occupational therapy, social counseling and initiation of follow-up care. CPR programs address past and present mental health problems and aim to prevent a relapse. Patients are supported in recognizing and changing dysfunctional patterns of behavior and relationships and in developing behavioral alternatives. In contrast to WMR programs, they do not integrate an explicit focus on work, work ability and return to work in the diagnostics and therapy. Rehabilitation follows the recommendations of the guidelines for inpatient rehabilitation centers concerning the medical rehabilitation of adults with psychosomatic and mental disorders [25, 26].

#### Intervention

Participants of the intervention group will receive a WMR program according to the guidelines for WMR [15, 27] as well as the current treatment standards and guidelines for the rehabilitation of mental disorders. WMR programs contain at least 11 h of work-related treatment modules [17]. As with the CPR programs, WMR programs follow a multimodal approach that comprises psychotherapy, psychoeducation, relaxation training, sports and exercise therapy, health education, expressive arts therapy, occupational therapy, social counseling and initiation of follow-up care. However, WMR programs more explicitly focus on work, work ability and return to work by including additional work-related diagnostics as well as work-related functional capacity training, work-related psychosocial groups and intensified social counseling. Table 1 describes the core modules in line with the TIDieR checklist [33].

**Table 1:** Description of core intervention modules according to the TIDieR checklist

*Brief name*	Work-related diagnostics	Social counseling	Work-related psychosocial groups	Work-related functional capacity training
*Why*	Work-related assessments are performed to plan and design therapeutic interventions individually. Diagnostic measures are also needed to develop recommendations for adapting the patient’s job environment.	The aims of social counseling in WMR are providing information about the various possibilities for supporting work participation and working out solutions for individual occupational and social problems.	The aim of work-related psychosocial groups is to learn how to deal with work-related conflicts.	The aim of work-related functional capacity training is to increase the capacity to cope job demands.
*What*	Work-related assessments determine individual rehabilitation needs by comparing work-related physical and psychosocial functional capacity with the patient’s job demands. Assessment of functional capacity and job demands is performed by interviews, tests and questionnaires. The rehabilitation team uses standardized profiling to contrast individual capacities and job demands [28–31]. The comparison of individual capacities and job demands reveals discrepancies in demands and capacities due to excessive demands and/or insufficient capacities.	Social counseling examines the individual work–life situation and provides socio-legal guidance and advice concerning further assistance within the social security system. The intervention usually takes place in the course of several counseling sessions. In the group session, presentation slides are used to illustrate possibilities available in the social security system. After individual consultation appointments, patients receive written summaries of the discussed contents.	The group discusses conflicts and interactional problems but also resources and skills that can be used in the workplace. The participants are taught knowledge about triggers. The exchange within the group enables the participants to work out strategies for coping with conflicts together. Additionally, preventive measures to avoid stress and conflicts are taught. Both psychoanalytical and behavioral therapy approaches are used.	In work-related functional capacity training, complex work routines relevant to the workplace are trained (e.g. group project work to explore interactive behavior).
Who provided	Therapists of different occupational backgrounds are involved, particularly physicians, psychologists, occupational therapists, exercise therapists and social workers.	Social counseling is provided by social workers.	Psychosocial sessions are performed by clinical psychiatrists or psychological therapists.	The intervention is primarily carried out by occupational therapists. In addition, parts of the training are carried out by physiotherapists, vocational trainers, psychologists and psychotherapists.
*How*	Diagnostic measures are performed face-to-face and individually.	The intervention consists of face-to-face individual sessions and an additional group intervention with a maximum of 15 participants.	Work-related psychosocial groups are conducted face-to-face in groups of a maximum of 15 people.	Work-related functional capacity training is performed face-to-face as a group intervention with a maximum of 15 participants.
*Where*	All treatment components are delivered in inpatient rehabilitation facilities. Lecture halls with screens for the lectures and the presentation of slides are available. Individual discussions take place in therapy rooms. Group therapy rooms are available for group therapy. Occupational therapy facilities are available and contain all the materials needed for the work-related functional capacity training.			
*When and how much*	The intervention is performed at the beginning and, if necessary, also during or at the end of the rehabilitation program for a total of at least 90 min.	Social counseling in the context of WMR is provided at least twice, which amounts to at least 30 min.	Sessions are scheduled four to ten times during a WMR program. Each session lasts at least 45 min, which corresponds to a minimum therapy dose of 180 min.	The work-related functional capacity training takes place at least six times, amounting to at least 360 min.
*Tailoring*	Work-related diagnostics is provided to all participants of WMR.	All participants of WMR are required to attend the individual appointment with a social worker. Participation in the additional lectures is determined according to individual needs.	All participants of WMR are required to attend work-related psychosocial groups.	All participants of WMR are required to attend work-related functional capacity training.
*How well*	All treatment components will be documented in the standardized rehabilitation discharge letters to assess the actual delivered dose. The rehabilitation teams use the corresponding codes of the classification of therapeutic interventions [32] developed by the German Pension Insurance for quality assurance in rehabilitation. In addition, patients will be asked about content and achievement of therapy goals at the end of the rehabilitation program with a standardized set of questions.			

### Outcomes and other measures

This study will assess one primary outcome as well as secondary outcomes and moderator variables. Outcomes and other measures will be assessed with patient questionnaires or extracted from the rehabilitation discharge letters. A complete list of all measured constructs, measurement points and expected scaling is shown in Table 1.

### Primary outcome

The primary outcome of this study is stable return to work 12 months after rehabilitation. Return to work will also be assessed at the three-month follow-up. Stable return to work has been defined in accordance with Kuijer and colleagues [34] as a minimum of 4 weeks of employment without sick leave at follow-up.

### Secondary outcomes

Secondary outcomes cover several dimensions of health, functioning and coping strategies (see below) and will be measured at all four measurement points. At the first follow-up after completing the rehabilitation program, participants will also appraise how the rehabilitation programs dealt with work-related issues and how satisfied they were with the treatment.

#### Employment

To cover participation in working life, the employment status (employed vs. unemployed) is noted. We record the professional position and economic sector. Moreover, we will assess if patients are on sick leave and how long they have been on sick leave. To assess the duration of sick leave, participants will be asked to report the number of weeks they have been off work for health reasons since discharge from the rehabilitation center. At baseline, this question is related to the last 12 months.

#### Self-reported work ability

Work ability will be assessed by the Work Ability Score (WAS), which is the first item of the Work Ability Index (WAI) [35] and compares current work ability with the lifetime best. The 11-point scale ranges from 0 (complete incapacity to work) to 10 (lifetime’s best work ability). The WAS is highly correlated with the overall WAI score [36].

#### Health-related quality of life

The 36-Item Short-Form Health Survey (SF-36) will be used to assess the health-related quality of life [37]. The SF-36 comprises eight subscales: physical functioning, physical role function, physical pain, general health perception, vitality, social functioning, emotional role function and psychological well-being. Each score lies in the range 0–100 points, with higher values indicating better health-related quality of life.

#### Psychosocial health

Psychosocial health will be measured by three modules of the German survey “HEALTH-49” (Hamburger Module zur Erfassung allgemeiner Aspekte psychosozialer Gesundheit für die therapeutische Praxis) [38]. The first module consists of three scales with a total of 18 items and assesses psychological and somatoform symptoms. The three subscales are somatoform disorders (7 items), depression (6 items) and phobic anxieties (5 items). Patients will be asked if they suffered from different symptoms such as back pain, headache or hopelessness in the last 2 weeks. All items were five-point graded (0 = not, 1 = little, 2 = medium, 3 = quite, 4 = much). The second module covers interactional difficulties. Patients will be asked if they had difficulties in interactional situations (e.g. “showing feelings to other people”) in the last 2 weeks. The module consists of one scale with seven items that were also five-point graded (0 = not, 1 = little, 2 = medium, 3 = quite, 4 = much). The third module assesses self-efficacy. With five items patients will be asked to state to what extent they feel able to do tasks, deal with strains or do things that are important to them despite their complaints. All items of the five-point scale from 0 (completely disagree) to 4 (completely agree) will be reversed afterwards. In each case the total score is the unweighted mean of all items, with higher values indicating higher impairments.

#### Job-related anxieties

A short 13-item form of the Job Anxiety Scale [39, 40] will be used to assess panic and fear regarding the workplace. Patients will be asked how much they agree with a set of situations, thoughts and feelings that can be experienced in connection with the workplace, e.g. “When I think about my workplace, I notice how everything inside me is tensed”. Responses range from 0 (completely disagree) to 4 (completely agree). All item scores will be averaged.

#### Regeneration and resistance beliefs

In order to assess attitudes and beliefs about coping with stressful events, a 20-item German questionnaire (Resistenzorientierung-Regenerationsorientierungs-Skala, ReRe) will be used [41]. Ten items each are used to assess the extent to which patients prefer coping strategies that focus on either recovery or endurance. Responses range from 1 (completely disagree) to 5 (completely agree). Individual items will be averaged to gain a regeneration score and an endurance score. Higher scores indicate higher regeneration or endurance.

#### Received dose of work-related treatment components

To assess the fidelity of our intervention according to the patients treated, we will employ a slightly modified version of a previously used set of items from a study that investigated the nationwide implementation of the WMR guidelines in patients with musculoskeletal disorders [42]. Participants report on 12 binary items as to whether they received WMR content in their rehabilitation program. Scores are aggregated to a total score of 0–12 points. This score reflects the content of the work-related components. Additionally, six items assess the perceived consistency of the work-related approach (e.g. the experience of a coherent return-to-work strategy). These items are five-point graded and scores are summed to a total score of 0–24 points. Finally, the achievement of work-related goals will be assessed by eight items that are also five-point graded and scores are aggregated to a total score of 0–32 points.

#### Treatment satisfaction

Treatment satisfaction will be assessed using the German version of the Client Satisfaction Questionnaire (CSQ-8) [43]. This questionnaire comprises eight items designed to assess various aspects of the patient’s satisfaction with treatment. Responses are four-point graded and the overall score lies in the range 8–32 points.

### Other measures

Additional measures will be assessed as potential effect modifiers and to conduct a health economic analysis.

#### Delivered dose of work-related components

Therapeutic interventions will be extracted from the standardized rehabilitation discharge letters [44]. The documentation for the therapeutic interventions will indicate adherence to the WMR guidelines. The delivered intervention dose is also a potential effect modifier.

#### Socio-medical assessment of capacity

Capacity for the most recent occupational activity and the general labor market, as well as recommendations for subsequent benefits, is assessed by the rehabilitation center. We take the information from the rehabilitation discharge letters [44].

#### Socio-demographic data

We will ask participants for socio-demographic data (age, gender, native language, educational level, partnership and children).

#### Use of medical and non-medical health care services

For our health economic evaluation we use the German Questionnaire for Health-Related Resource Use in an Elderly Population (Fragebogen zur Inanspruchnahme medizinischer und nicht-medizinischer Versorgungsleistungen im Alter, FIMA) [45]. We will ask about the type of health insurance, the medication taken in the last 7 days, outpatient visits to the doctor and therapeutic services used in the last 3 months, rehabilitation measures, outpatient operations or stays in day clinics and inpatient treatment in the last 12 months. We will evaluate the answers with calculated unit costs [46]. See Table 2 for measures, assessment, expected scaling and maesurement occasions.

**Table 2:**
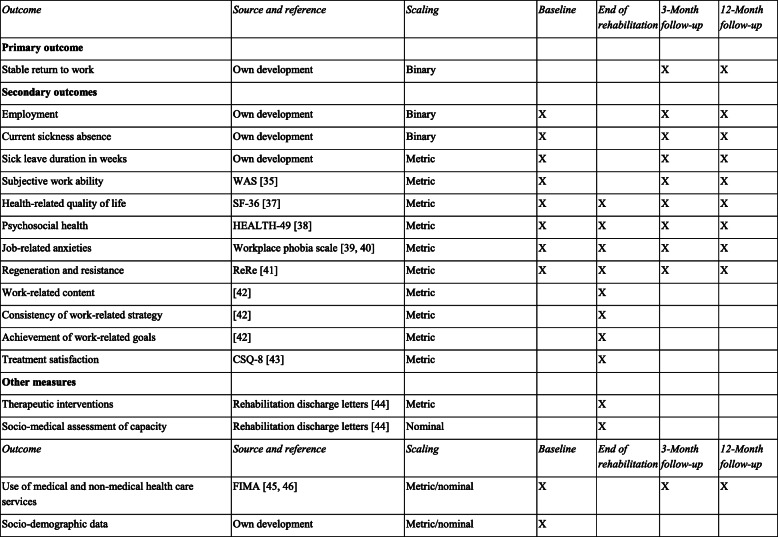
Schedule of enrolment, interventions and assessments

### Participant timeline

Participants fill in the baseline questionnaire at the beginning of rehabilitation. At the end of rehabilitation the participants fill in the first follow-up questionnaire at the rehabilitation center. At the 3- and 12-month follow-ups, participants who completed the baseline questionnaire will receive follow-up questionnaires from the University of Lübeck. Focus groups with some of the participants will be conducted during the intervention as well as three and 12 months after rehabilitation (see Table 3 for a full schedule of enrolment, interventions and assessments).

**Table 3:**
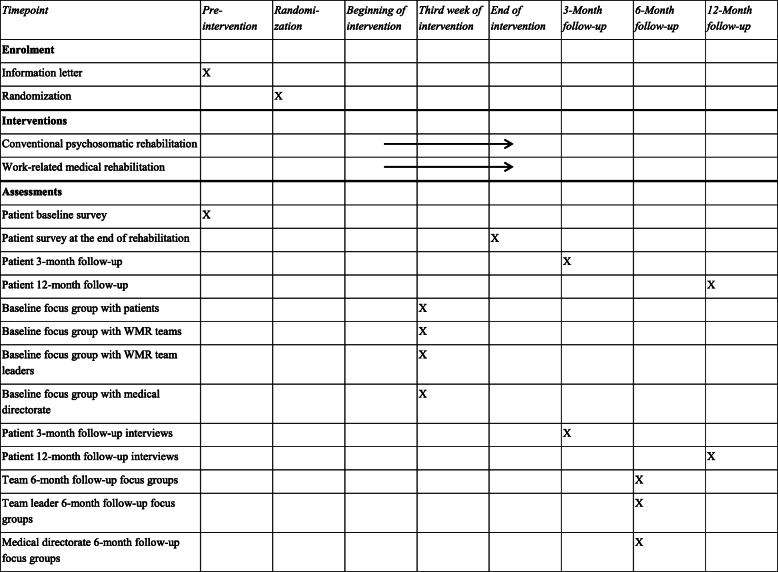
Schedule of enrolment, interventions and assessments

### Sample size estimation

**Fig. 1:**
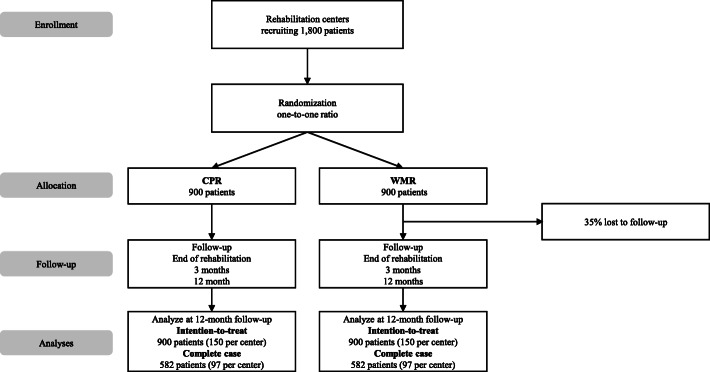
Flow of participants

In order to ensure a 20% increase in the proportion of rehabilitants with stable return to work per rehabilitation center (CPR: 40%; WMR: 60%; power: 80%; level of significance: 5%), an analysis sample of 194 persons (i.e. 97 persons per intervention arm) is necessary. Although we will use multiple imputations to perform an intention-to-treat analysis, we increase the sample size to compensate for the potential loss of participants during our follow-up assessments. This ensures sufficient power even if only complete cases are analyzed. Assuming a response rate of 65% after 1 year, we will recruit 300 patients per rehabilitation center. In a total sample of 1800 patients, a minimal difference of seven points can be detected. In a total sample of 1164 patients (response rate of 65%), a minimal difference of eight points can be detected (Fig. 1).

Of the 1800 persons recruited, eight patients from each of the intervention and control groups are supposed to take part in interviews for a panel study. The patients should be in the third week of their rehabilitation program. The second and third follow-up interviews will be conducted with three persons from these focus groups. The focus groups with the team members are planned with five persons per rehabilitation center. The number of participants in the focus groups with the team leaders will depend on the number of teams per rehabilitation center.

### Recruitment

The rehabilitation centers check all newly arrived rehabilitation patients with mental disorders between the ages of 18 and 60 years to see whether they have an increased risk of not returning to work. This will be detected using the SIMBO-C [24]. In the case of a positive result (i.e. a score of at least 27 points), the study assistant informs the person about the trial. The study assistant hands out the study documents to the patient. An information letter details the content and objectives of the study as well as the patient’s personal rights regarding the handling of personal data. In case of participation, the patients endorse informed consent and complete the baseline questionnaire. If one of the follow-up questionnaires is not returned, a questionnaire will be sent again, with a reminder to all participants after 3 weeks.

Recruitment for the interviews will be carried out by the study assistants in the rehabilitation centers. Informed consent forms are handed out during the recruitment process and can be discussed with the person carrying out the interview if necessary and will be signed before the first interview. However, the teams, team leaders and medical directorates will be recruited by the person conducting the interviews.

### Allocation

A separate randomization sequence is created for each rehabilitation center by the University of Lübeck. For the computer-generated randomization lists, blocks of four and eight are combined in order to guarantee balanced case numbers, even if the lists cannot be processed completely. The randomization envelopes are sealed and will be opened in the rehabilitation centers after informed consent of the participants is determined. After arriving at one of the six study centers, the potential study participants are informed in detail about the study, both orally and in writing, and are asked to participate by the study assistants, who have been trained by the research team. In particular, the potential participants are informed that there will be two different study groups (intervention group and control group) and that allocation to the two groups will be randomized.

### Blinding

No one will be blinded before, during or after the trial as the realized rehabilitation program will be recognizable for all stakeholders.

### Data collection

Outcomes and other measures will be assessed with patient questionnaires or will be extracted from the rehabilitation discharge letters (see Table 2). For participants who do not complete follow-up questionnaires, missing data will be imputed. If participants withdraw their participation, the collected data will be deleted.

Three consecutive interviews with participants of the complementary panel study are planned. The first interviews will take place during rehabilitation in the rehabilitation centers as focus groups. After three and 12 months, follow-up interviews with the same sample will be conducted as individual interviews by telephone or video call. Furthermore, members of the multi-professional teams, team leaders and the respective medical directorate of each rehabilitation center will be interviewed. Again, the first interviews will be conducted in the rehabilitation centers and the second interview after 6 months by telephone or video call. The interviews of teams and team leaders will be conducted as focus groups.

### Data management

Questionnaires will be entered manually into an electronic database by trained research assistants at the University of Lübeck. Data on the documented therapeutic dose are taken from the rehabilitation discharge letters and will also be entered into the electronic database. All personal data will be removed and replaced by the unique study identifier by the rehabilitation centers.

Recordings from the focus groups and interviews will be transcribed by trained assistants at Nordhausen University of Applied Sciences. The names of patients and staff will be pseudonymized during the transcription process. These pseudonyms will be added to the study list. Finally, questionnaire, administrative and qualitative data can be linked by the unique study identifier. Data management will be carried out by the authors of the protocol. Data access is limited to the authors and to the research assistants in the research team.

### Statistical methods

#### Treatment effect

We use random-effects models to take into account that different true effects are possible in different rehabilitation centers. Linear models will be used for continuous outcomes and logistic models for binary outcomes. Baseline scores of outcomes will be included as covariates. Possible differences in effects at the level of the rehabilitation centers may be associated with WMR or CPR implementation. In order to test an effect modification due to the implementation of either WMR or CPR, subgroups are formed based on the received treatment dose (content, consistency, achievement of objectives) and the work-related treatment dose delivered. To reduce the number of subgroup analyses, the mean differences between WMR and CPR for dose delivered and dose received are used to categorize the subgroups (high distinguishability vs. low distinguishability). We assume that greater distinguishability of both interventions based on treatment dose delivered and received is associated with higher effects. Further characteristics of the rehabilitation centers for subgroup analyses may derive from the focus groups. Gender, age, migration background and education are examined as possible moderating patient characteristics. Multiple imputations will be used to fill in missing data and to perform an intention-to-treat analysis.

#### Health economic evaluation

An incremental cost–utility analysis is performed [47]. Costs are recorded from the perspective of society as a whole [46]. For the evaluation of resources according to Seidl et al. [45], the calculated unit costs proposed by Bock et al. [46] are used. The recording of quality-adjusted life years for the evaluation of health benefits is conducted according to Brazier et al. [48].

### Focus groups and interviews

Two central aspects will be examined in the qualitative part. On the one hand, we will identify personal and environmental facilitators and barriers [49], which influence a successful return to working life. On the other hand, the implementation of WMR as well as its further development and subsequent adaptions of the program’s core components implementation process. This will be explored against the background of the implementation model “Consolidated Framework for Implementation Research” [50].

For these two interview areas, two research questions are guiding. First, which characteristics of the patient and which environmental factors influence a successful return to working life after psychosomatic WMR? Second, which characteristics influence a successful implementation and realization of WMR?

The study will be conducted as a qualitative longitudinal study. Data will be collected using guided interviews [51]. The interviews will be conducted by one of the authors (NG).

A maximum of eight patients (each intervention group and control group) from the six participating rehabilitation facilities will be interviewed three times. Patients already participating in the quantitative study will be eligible for participation. The first interview will be conducted in the clinics as a focus group. We will start with discussing personal and environmental factors, then we will address the implementation of WMR and its core components. The second and third interview will each be conducted by telephone or video conference 3 months and 12 months after the end of rehabilitation. We will include three patients from each focus group for these follow-up interviews. The guidelines of the follow-up interviews are similar to the initial guidelines, but their content is adapted to the home situation to be able to explore the individual course after rehabilitation.

In addition, one team per rehabilitation facility, all team leaders and the medical directors are interviewed. Two interviews are planned with these participants. The first appointment will be conducted in person in the rehabilitation facility. The second interview, 6 months later, will be held by telephone or video conference. All interviews with teams and team leaders are conducted as focus groups. The interviews of the teams, team leaders and medical directors broach the same broad two areas as those of the patients. However, the patients’ situation will be assessed from a professional perspective. In addition, the working conditions of the therapeutic staff in the rehabilitation facilities will be addressed in these interviews.

The follow-up interviews are done to be able to capture changes and progress. A pretest is planned to clarify the feasibility and appropriateness of our interview questions. We will adapt our final guide accordingly. All interviews are transcribed [52] and analyzed by content analysis [53]. We will use quality criteria for quality research to outline the applicability of our findings [53].

### Monitoring

A data and safety monitoring board accompanies the study. The committee consists of four statisticians and psychologists who attend the advisory board meetings, have insight into the statistical methods and provide advice to the university researchers. The committee provides feedback on the current status of the study and on already available results. The feedback is given orally at the advisory board meetings or in writing when milestones are reached. Members of the data and safety monitoring board are independent of the sponsor and have no competing interests.

The study assistants in the rehabilitation centers, who were recruited and trained specifically for the study, provide the universities with feedback on spontaneously reported adverse events and other unintended effects of trial interventions or trial conduct. The universities then document this information.

## Discussion

This study is intended to provide evidence of the relative effectiveness of a complex, newly implemented work-related rehabilitation strategy for patients with mental disorders. We will provide a detailed description of the programs on our website: www.mbor-psychosomatik.de. The findings of this study will be published in peer-reviewed journal articles and conference presentations.

The study protocol has been prepared according to the SPIRIT checklist (Standard Protocol Items: Recommendations for Interventional Trials) [54].

## Supplementary Information

**Additional file 1.** Participant information letter.

## Data Availability

Not applicable.

## Abbreviations

CPR: Conventional psychosomatic rehabilitation
FIMA: Questionnaire for Health-Related Resource Use in an Elderly Population (Fragebogen zur Inanspruchnahme medizinischer und nicht-medizinischer Versorgungsleistungen im Alter)
GPI: German Pension Insurance
ReRe: Resistance and Regeneration Orientation Scale (Resistenzorientierung-Regenerationsorientierungs-Skala)
SF-36: 36-Item Short-Form Health Survey
WAI: Work Ability Index
WAS: Work Ability Score
WMR: Work-related medical rehabilitation
