# Supplementary material for: Work-related medical rehabilitation in patients with mental disorders: the protocol of a randomized controlled trial (WMR-P, DRKS00023175)
Source: BMC Psychiatry. 2021 May 3;21:225. doi: 10.1186/s12888-021-03181-7 (PMC8091693; doi:10.1186/s12888-021-03181-7)
Supplement: Supplementary file 1 — Additional file 1. Participant information letter. [file 12888_2021_3181_MOESM1_ESM.docx]

## Additional File 1: Participant information letter

Dear Patient,

With this letter we inform you about our study: "Work-related medical rehabilitation in patients with mental disorders".

The study consists of two parts. In the first part the University of Lübeck surveys the participants by using questionnaires to answer in written form. In the second part, Nordhausen University of Applied Sciences conducts research interviews with some of the participants. This information letter informs you about the written survey.

We – the researchers of the University of Lübeck and Nordhausen University – would like to invite you to participate in this study. Based on the following information, you can decide if you would like to participate. Feel free to keep this information letter.

1. Who is responsible for this study?

The head of the study is Prof. Dr. Matthias Bethge of the University of Lübeck (Institute for Social Medicine and Epidemiology, Section for Rehabilitation and Work, Ratzeburger Allee 160, 23538 Lübeck). Any questions relating to this project and the questionnaires should be directed to Miriam Markus of the University of Lübeck (Tel.: 0451/9299-5118; e-mail: miriam.markus@uksh.de). Mrs Markus will be able to answer any questions you may have about this study.

Your direct contact in the rehabilitation center is: […]

This research is funded by the Federal German Pension Insurance.

2. What is the aim of our study?

Within psychosomatic rehabilitation, two different treatment programs are currently used: medical rehabilitation and work-related medical rehabilitation. It is not clear whether one of the programs leads to better results than the other, therefore our study aim is to compare the effectiveness of both treatment programs.

3. How does the study proceed? What can you do?

For this study you will be randomly assigned to one of the two treatment programs.

Your contact person will pick a sealed envelope in which the form of rehabilitation is noted. To check if one of the programs leads to better treatment results than the other, we conduct written surveys. Four surveys in total are planned for each participant: at the beginning and at the end of the rehabilitation program, as well as three and 12 months after completion of your program.

The questionnaires focus, for example, on your state of health, your functional capacity, your use of health care and your treatment satisfaction. The first questionnaire is 13 pages long but the subsequent questionnaires are much shorter.

If you wish, you can view the questionnaires before you agree to participate in this study. For this, feel free to speak to your above-mentioned contact person at your rehabilitation facility. On average it will take you about 30 minutes to complete the questionnaire.

In addition to the information you provide in the questionnaires, we would like to use study-relevant information from your medical discharge report. This will contain a list of the diagnostic and therapeutic services you received, information about the treatment diagnosis and ability to work, the socio-medical performance assessment and the medical recommendations for further treatment, as well as your year of birth and gender.

4. How will your data be processed? Who will get to know the names of the participants?

After you agree to participate in our study, your name and address will be registered in a participant list by your rehabilitation facility and you will be given a study number; this study number will be on your questionnaires and your discharge report. This procedure is called pseudonymization and ensures that we cannot draw any conclusions from the research data that identify a participant. Your name will not appear on the questionnaires or the provided data from your discharge report. The study number is required to link the questionnaire data with the data from the discharge report. Unlike your name, this number is very important to us because it enables us to interpret the data. The participant list will be destroyed by your rehabilitation center by 31 December 2023 at the latest. Subsequently, names and addresses of study participants will be anonymized and will no longer be available after the study.

We, as the researchers, get a copy of the study participant list for sending the questionnaires to the attendees three and 12 months after the end of rehabilitation. This list will not be used by us for any other purpose than for posting the questionnaires. We will store the list separately to the research data and it will be password protected. Once the questionnaires have been posted, the list will be destroyed. This procedure ensures that no one can link the research data with your name.

For your reassurance, we are only permitted to use your data with your clear, free and signed permission. All researchers are obliged to treat your data in a strictly confidential manner. The data protection officer of the University of Lübeck is […]

5. What will my pension insurance get to know?

Your pension insurance receives no information regarding your participation or non-participation. Also, your pension insurance does not receive any personal data from the study. All information that you provide in the written surveys will be processed in a strictly confidential and anonymous manner at the University of Lübeck.

6. Voluntary principle

Your participation in our study is completely voluntary. Your data will only be used if you have signed the consent form. If you do not wish to participate, you do not need to hand in your consent. Additionally, it is possible to withdraw your consent at any time and without giving a reason. If this should be the case, you just need to inform the above-mentioned contact person in your rehabilitation center. We will then make your name unrecognizable in the list of participants.

Furthermore, we will delete your data collected for the research if assignment is possible. If you want to withdraw your consent but your rehabilitation treatment has already ended, you can get in touch with the above-mentioned contact person at the University of Lübeck. If identification is still possible, we will delete your data.

If you decide not to participate in this study, your rehabilitation center will choose the treatment program for you. The assignment to one of the programs is then not random and you will not receive any questionnaires.

7. Request for participation

If you have read the above information, understand the content and you would like to take part in the study, we ask that you sign two consent forms. One of these consent forms needs to be given to the above-mentioned contact person in your rehabilitation center. The other consent form is for you. Please keep this form and also this letter for future reference.

We would be very grateful if you decide to participate in our study. The more people who take part, the more reliable the results will be.
